# Isolated Medial Ankle Tenosynovitis of the Flexor Digitorum and Posterior Tibialis Tendons: A Case Report of the Role of Early Musculoskeletal Ultrasound in Identifying Anti-citrullinated Protein Antibody (ACPA)-Positive At-Risk Rheumatoid Arthritis

**DOI:** 10.7759/cureus.116076

**Published:** 2026-09-10

**Authors:** Abira A Chowdhury, Wan-Ying Lin, Sissmol Davis, Mohammad Hasibul Chowdhury, Chathura Wijewardena

**Affiliations:** 1 Internal Medicine, University at Buffalo, Buffalo, USA; 2 Internal Medicine, Jalalabad Ragib Rabeya Medical College, Sylhet, BGD; 3 Rheumatology, Buffalo Rheumatology, Buffalo, USA

**Keywords:** ankle pain, anti-ccp antibodies, early rheumatoid arthritis, erosive disease, musculoskeletal ultrasound, mutated citrullinated vimentin, rheumatoid arthritis, seropositive arthritis, tenosynovitis

## Abstract

Tenosynovitis is a recognized feature of rheumatoid arthritis (RA), yet it typically presents alongside symmetric polyarticular synovitis. Isolated focal tenosynovitis as the primary and initial manifestation of RA is uncommon and frequently leads to diagnostic delays. We present the case of a 62-year-old female who presented with localized right ankle pain in the absence of systemic joint involvement or elevated inflammatory markers. While the rheumatoid factor was negative and both the erythrocyte sedimentation rate and C-reactive protein levels were within normal limits, musculoskeletal ultrasound revealed focal tenosynovitis of the flexor digitorum and posterior tibialis tendons, accompanied by possible early erosive changes of the medial malleolus. Markedly elevated titers of anti-cyclic citrullinated peptide (112 U) and mutated citrullinated vimentin (171 U) antibodies supported the diagnosis. The patient responded rapidly to prednisone and was subsequently transitioned to methotrexate. This case emphasizes that isolated tenosynovitis can be the early presenting feature of seropositive RA. It underscores the critical role of advanced serologic testing and high-resolution imaging in identifying early-stage disease when traditional clinical markers are absent, allowing for timely intervention to prevent irreversible structural damage.

## Introduction

Rheumatoid arthritis (RA) is a chronic systemic inflammatory disease characterized by persistent synovial inflammation that can lead to progressive joint destruction and functional disability. Although joint pain, swelling, and prolonged morning stiffness are hallmark clinical features, periarticular manifestations, including tenosynovitis, are also well recognized [1].

Tenosynovitis, resulting from inflammation of tendon sheaths, is frequently observed in RA but typically occurs in conjunction with synovitis or established polyarticular disease. Isolated tenosynovitis as the sole presenting manifestation is uncommon and may delay recognition of an RA-associated inflammatory process [2].

Recent advances in imaging have demonstrated that subclinical tenosynovitis may represent one of the earliest detectable inflammatory abnormalities in individuals with RA-related autoimmunity and may precede clinically apparent arthritis [3]. Importantly, the 2010 American College of Rheumatology/European League Against Rheumatism (ACR/EULAR) classification criteria for RA require definite clinical synovitis in at least one joint as an entry requirement for classification as definite RA [4]. Therefore, patients with anti-citrullinated protein antibody (ACPA) positivity and inflammatory tenosynovitis but without clinical synovitis may represent an at-risk or early RA-spectrum phenotype rather than meeting the classification criteria for established RA. Early recognition of inflammatory musculoskeletal manifestations remains important because timely evaluation and treatment may reduce the risk of persistent inflammation and structural damage.

We report a case of ACPA-positive inflammatory tenosynovitis involving the flexor digitorum and posterior tibialis tendons of the medial ankle without clinically apparent synovitis or systemic inflammation, illustrating the potential role of musculoskeletal ultrasound in identifying an early RA-associated phenotype.

## Case presentation

A 62-year-old woman with a past medical history significant only for well-controlled hypertension and hyperlipidemia was referred for evaluation of persistent right ankle pain of two months’ duration. The pain developed insidiously and was localized to the medial ankle. The patient described moderate pain associated with approximately one hour of morning stiffness confined to the affected ankle. Over time, she noted subjective swelling in the same region.

She denied previous episodes of joint pain or swelling involving the ankle or any other joints. There was no history of trauma, overuse injury, recent infection, or preceding illness. Over-the-counter analgesics, including acetaminophen and ibuprofen, provided partial relief. Despite the discomfort, she remained able to ambulate and bear weight without significant functional limitation.

The patient denied systemic symptoms, including fever, night sweats, weight loss, or lymphadenopathy. Review of systems was otherwise unremarkable. She was taking atorvastatin 20 mg daily for hyperlipidemia. Statin-associated muscle symptoms were considered during the evaluation; however, the patient had no muscle weakness, and her symptoms were specifically localized to the medial right ankle. A creatine kinase (CK) level was not obtained at the time of evaluation.

On physical examination, the range of motion of all major joints was preserved and appropriate for age. Examination of the right ankle revealed mild warmth over the medial aspect with focal tenderness around the medial malleolus. No visible swelling or erythema was noted. Range of motion of both the tibiotalar and subtalar joints was preserved and symmetrical bilaterally. Pain was reproduced with resisted inversion of the ankle, suggesting involvement of the medial ankle tendon complex, particularly the posterior tibialis and adjacent flexor tendons.

Initial laboratory testing showed no evidence of systemic inflammation. Rheumatoid factor (RF), erythrocyte sedimentation rate (ESR), and C-reactive protein (CRP) were within normal limits. The complete blood count was normal, with no evidence of leukocytosis. We did not perform a myositis panel because no muscle weakness was noted.

However, additional serologic testing revealed markedly elevated RA-associated autoantibodies, as detailed in Table 1. 

**Table 1:** Initial laboratory evaluation and serologic profile.

Laboratory parameter	Patient value	Reference range	Interpretation
White blood cell (WBC) count	7.4 x 10³/µL	4.0-11.0 x 10³/µL	Normal
Platelet count	245 x 10³/µL	150-450 x 10³/µL	Normal
Hemoglobin	13.2 g/dL	12.0-15.5 g/dL	Normal
Erythrocyte sedimentation rate (ESR)	12 mm/hour	0-20 mm/hour	Normal
C-reactive protein (CRP)	3.2 mg/L	<5.0 mg/L	Normal
Rheumatoid factor (RF)	<10 IU/mL	<14 IU/mL	Negative
Antinuclear antibody (ANA)	Negative	Negative	Negative
Anti-cyclic citrullinated peptide	112 U	<20 U	Markedly elevated
Mutated citrullinated vimentin	171 U	<20 U	Elevated

Point-of-care musculoskeletal ultrasound was performed by a RhMSUS-certified rheumatologist. The right ankle demonstrated focal tenosynovitis involving the flexor digitorum tendon, corresponding to the region of maximal tenderness, with tendon-sheath fluid and thickening, as well as hypoechoic peritendinous changes (Figure 1). Partial tenosynovial inflammation of the adjacent posterior tibialis tendon was also observed, indicating involvement of multiple medial ankle tendon structures (Figures 1-3). Power Doppler imaging demonstrated increased vascularity within the tendon and surrounding soft tissues, consistent with active inflammation (Figures 2-4). In addition, ultrasound demonstrated cortical irregularity of the medial malleolus, concerning for an early erosive change. These findings provided an anatomic correlate for the patient’s symptoms and favored inflammatory tenosynovitis over purely mechanical tendinopathy.

**Figure 1:**
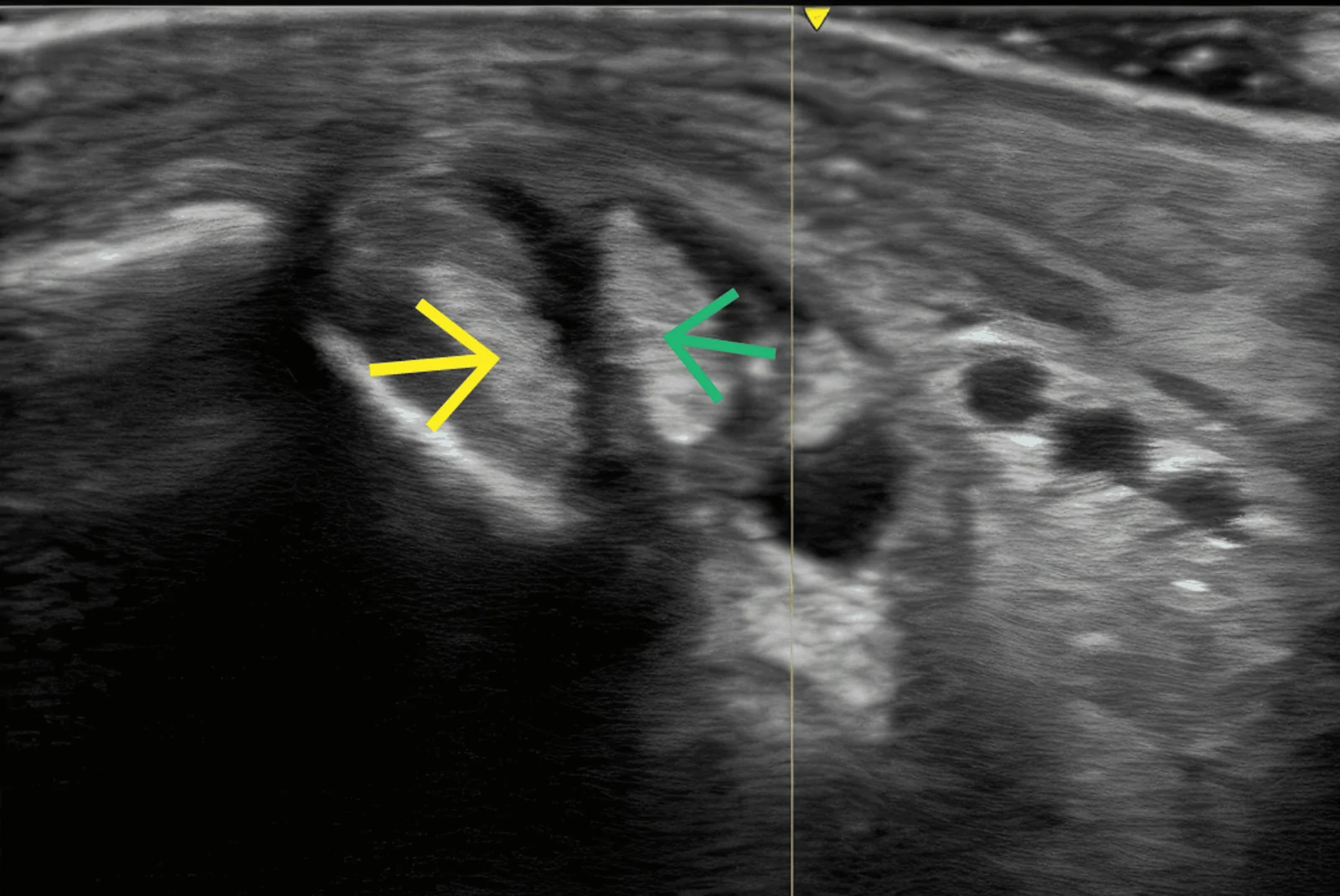
Transverse ultrasound view (short axis) of the right medial ankle. The tibialis posterior (yellow arrow) and flexor digitorum (green arrow) tendons are shown in short axis. Hypoechoic widening of the tendon sheaths adjacent to the tendons represents tenosynovial fluid and edema.

**Figure 2:**
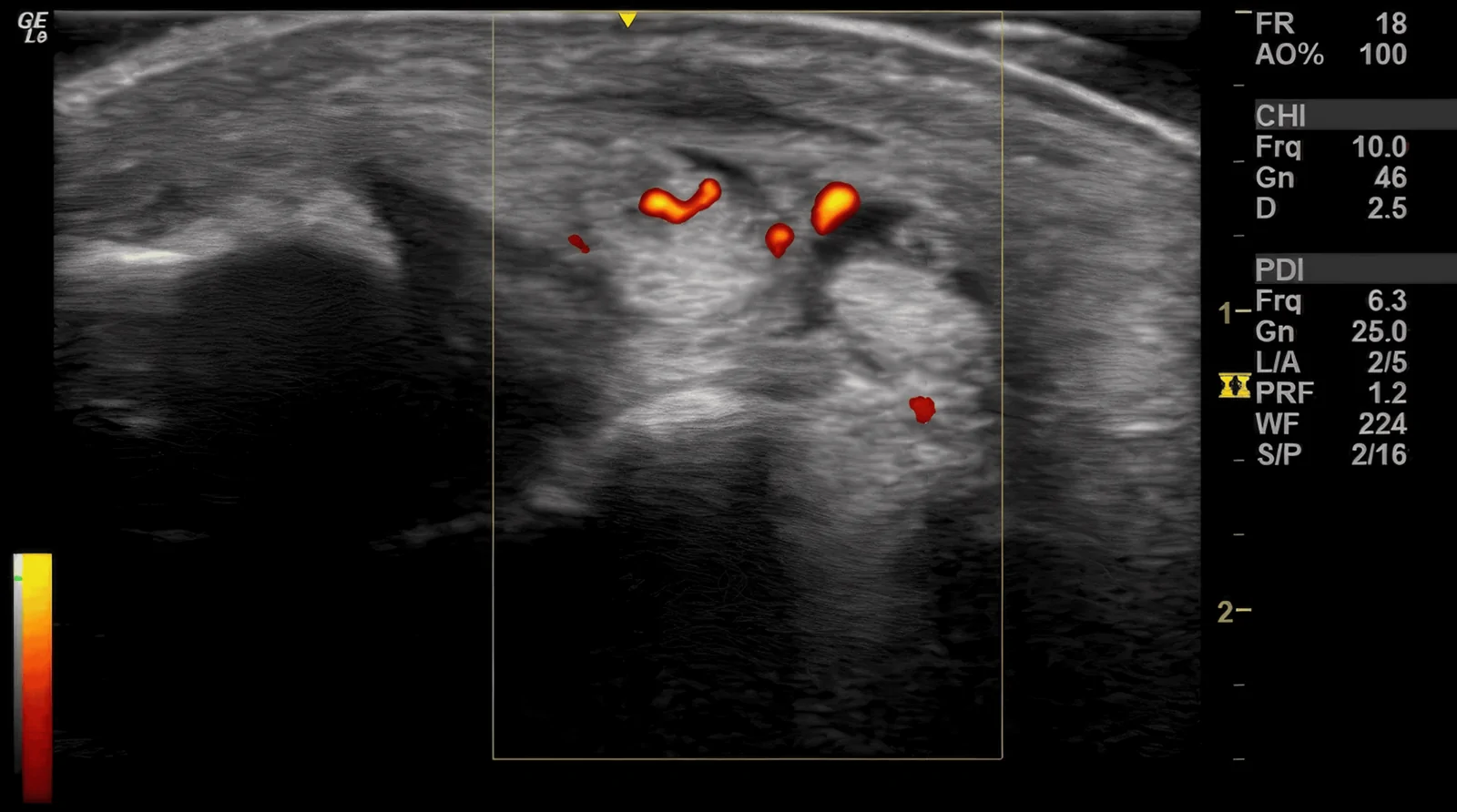
Transverse ultrasound view (short axis) with Power Doppler of the right medial ankle. Power Doppler signal is present within the posterior tibialis tendon and the hypoechoic tendon-sheath space surrounding the flexor digitorum tendon, indicating active tenosynovial inflammation.

**Figure 3:**
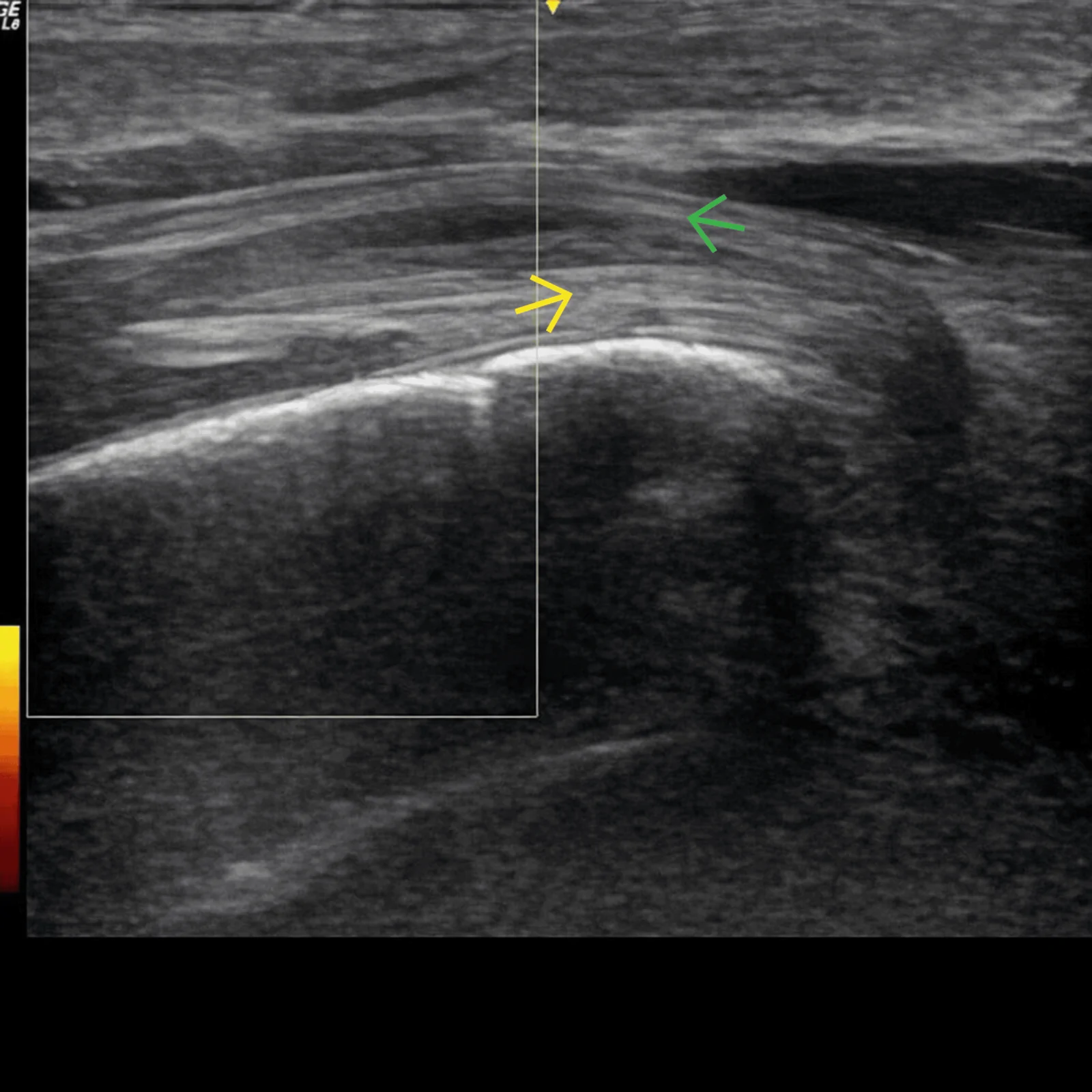
Longitudinal ultrasound view (long axis) of the right medial ankle (region within the box). From deep to superficial, the tibialis posterior (yellow arrow) and flexor digitorum (green arrow) tendons are shown. The hypoechoic area between the tendons and the anechoic area at the distal flexor digitorum tendon represent tendon-sheath fluid and edema.

**Figure 4:**
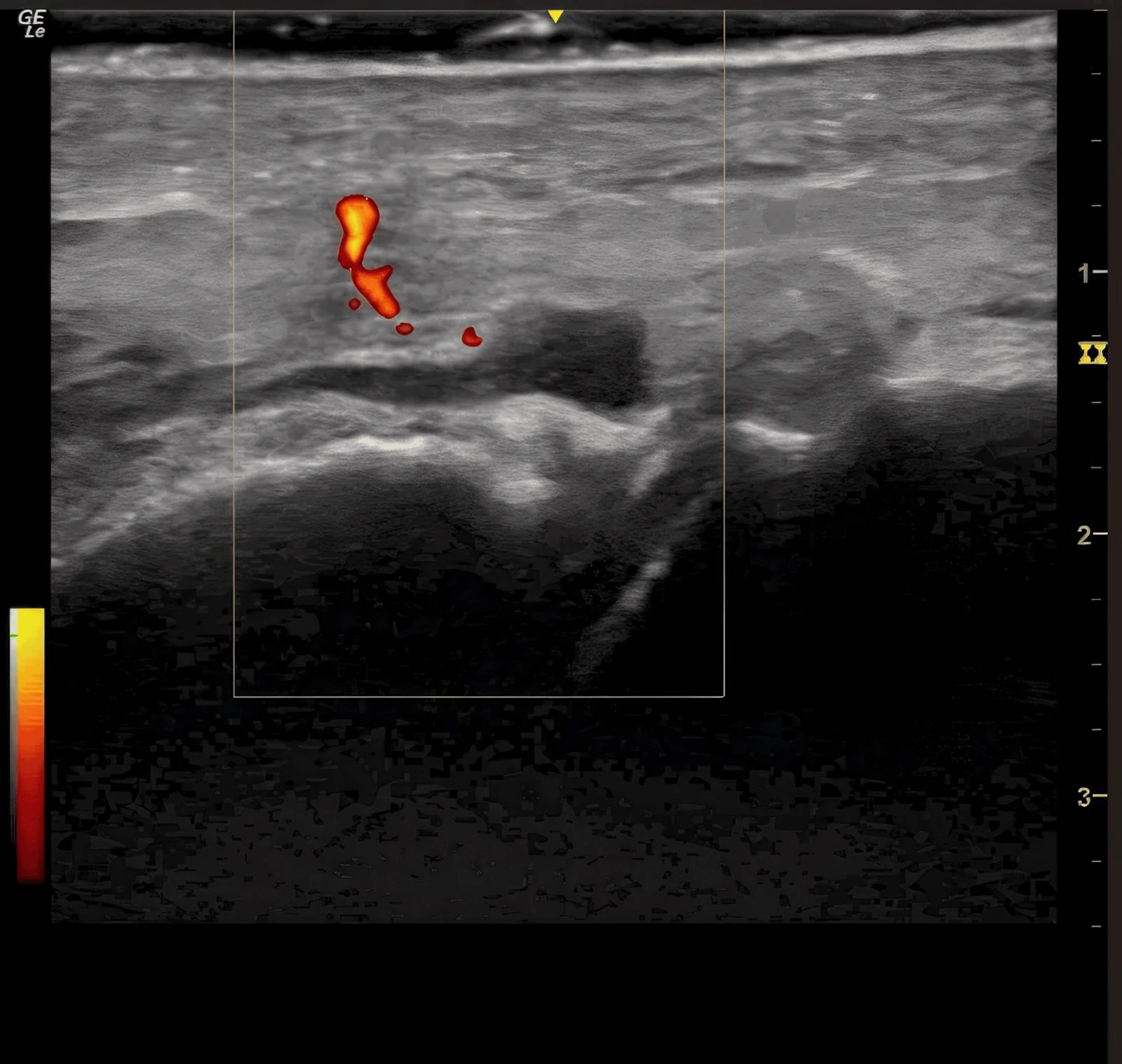
Longitudinal ultrasound view (long axis) with Power Doppler of the right medial ankle. The boxed distal peritendinous region shows Power Doppler signal, indicating inflammation, with an adjacent hypoechoic/anechoic area representing tendon-sheath fluid.

The patient did not meet the 2010 ACR/EULAR classification criteria for RA because she had no clinically swollen joints, which is a mandatory entry criterion. Based on the focal inflammatory tenosynovitis identified on examination and ultrasound, together with high-titer anti-cyclic citrullinated peptide (anti-CCP) and anti-mutated citrullinated vimentin (anti-MCV) antibodies, the working characterization was ACPA-positive inflammatory tenosynovitis consistent with early RA-spectrum disease.

The patient was initially treated with a tapering course of prednisone, consisting of 40 mg daily for 10 days followed by 20 mg daily for an additional 10 days, resulting in significant improvement in pain and morning stiffness.

Subsequently, methotrexate was initiated at 15 mg weekly with folic acid supplementation, resulting in sustained clinical improvement. During follow-up, an attempt was made to reduce the methotrexate dose to 10 mg weekly; however, ankle pain recurred within four weeks. Re-escalation to 15 mg weekly led to rapid resolution of symptoms. The clinical response to anti-inflammatory and disease-modifying therapy supported an underlying inflammatory arthritis phenotype, although treatment response alone was not considered diagnostic.

The patient has now been followed for approximately one year and remains clinically stable, with excellent symptom control on methotrexate 15 mg weekly. Notably, she has not developed clinical synovitis or inflammatory arthritis involving other joints.

## Discussion

This case highlights an uncommon presentation in which isolated medial ankle tenosynovitis involving the flexor digitorum and posterior tibialis tendons was the sole clinical manifestation of an ACPA-positive early RA-spectrum process. Although tenosynovitis is frequently observed in established RA, its occurrence as an isolated presenting feature - in the absence of clinically apparent synovitis - remains underrecognized. Applying the 2010 ACR/EULAR classification criteria, the patient would accrue only 4 of 10 points: 3 points for high-positive ACPA (anti-CCP >3× the upper limit of normal) and 1 point for symptom duration ≥6 weeks, with no points for joint involvement (no clinically swollen joints), normal acute-phase reactants, or negative rheumatoid factor. This score falls below the ≥6/10 threshold required for classification as RA. More fundamentally, because the criteria can be applied only to patients with at least one joint demonstrating definite clinical synovitis, our patient - who lacked clinically apparent synovitis - did not meet the entry requirement for classification. She is therefore more accurately characterized as having ACPA-positive inflammatory tenosynovitis within the at-risk/early RA spectrum rather than established rheumatoid arthritis [4].

Tenosynovitis in RA shares similar pathophysiologic mechanisms with joint synovitis, including synovial lining hyperplasia, infiltration by inflammatory cells - predominantly CD4^+^ T lymphocytes and CD68^+^ macrophages - and cytokine-mediated tissue damage [5]. These findings support the concept that tendon-sheath inflammation represents an integral component of early RA rather than a secondary phenomenon.

Increasing evidence suggests that tenosynovitis may precede clinically apparent arthritis. Imaging studies have demonstrated a high prevalence of tendon-sheath inflammation in ACPA-positive individuals before the development of overt arthritis [6]. In some cohorts, tenosynovitis has been frequently observed during the earliest phases of RA development and may serve as an imaging marker of progression risk.

Musculoskeletal ultrasound can detect tendon-sheath inflammation and early erosive change even when examination and inflammatory markers are normal. However, a definite ultrasound erosion requires a cortical break confirmed in two perpendicular planes; a single-plane cortical irregularity may reflect a physiologic bone channel or artifact. In this case, the suspected cortical irregularity of the medial malleolus was not confirmed in two planes and therefore represents a possible early structural change warranting follow-up imaging rather than a definite erosion [7].

Despite negative RF and normal inflammatory markers, the markedly elevated anti-CCP antibody level strongly supported an RA-associated autoimmune phenotype. Anti-CCP antibodies are highly specific for RA and are associated with an increased risk of persistent and erosive disease [8]. The additional elevation of anti-MCV antibodies further supported the autoimmune nature of the presentation. Combined testing for anti-CCP and anti-MCV may provide additional information in early or atypical presentations [9]. However, seropositivity alone does not establish definite RA without the clinical findings required for classification.

Recent trials have explored early intervention in individuals at risk of RA. The TREAT EARLIER trial enrolled patients with clinically suspect arthralgia and subclinical joint inflammation on MRI - including tenosynovitis as a qualifying imaging feature - but without clinically apparent arthritis, a phenotype that parallels our patient's ACPA-positive inflammatory tenosynovitis in the absence of clinical synovitis. In that trial, a time-limited course of methotrexate improved symptoms, physical functioning, and MRI-detected inflammation but did not prevent progression to clinical arthritis [10]. This supports recognizing isolated tenosynovitis as a potential early manifestation of RA-spectrum disease, in which early identification of high-risk serologic markers and timely therapy may reduce inflammation and limit structural damage.

## Conclusions

This case illustrates an uncommon presentation within the early seropositive RA spectrum, characterized by isolated medial ankle tenosynovitis involving the flexor digitorum and posterior tibialis tendons, with normal inflammatory markers but strongly positive anti-CCP and anti-MCV antibodies. Musculoskeletal ultrasound played a key role in identifying early inflammatory and erosive changes. Clinicians should consider RA in the differential diagnosis of unexplained focal tenosynovitis, particularly when high-risk ACPA are present, as early recognition and treatment are essential to prevent long-term joint damage.
